# New Tri-State Medical Society

**Published:** 1889-09

**Authors:** 


### NEW TRI-STATE MEDICAL ASSOCIATION.

It is proposed to organize a new Tri-State Medical Association, (Georgia, Tennessee and Alabama), and a call has been issued for a meeting in Chattanooga, Tennessee, the third Tuesday in October. Address Dr. F. T. Smith, secretary.

